# Proteomic and metabolomic changes driven by elevating myocardial creatine suggest novel metabolic feedback mechanisms

**DOI:** 10.1007/s00726-016-2236-x

**Published:** 2016-05-03

**Authors:** Sevasti Zervou, Xiaoke Yin, Adam A. Nabeebaccus, Brett A. O’Brien, Rebecca L. Cross, Debra J. McAndrew, R. Andrew Atkinson, Thomas R. Eykyn, Manuel Mayr, Stefan Neubauer, Craig A. Lygate

**Affiliations:** 1 Division of Cardiovascular Medicine, Radcliffe Department of Medicine, and the BHF Centre of Research Excellence, University of Oxford, Oxford, UK; 2 King’s British Heart Foundation Centre, King’s College London, London, UK; 3 Division of Imaging Sciences and Biomedical Engineering, King’s College London, London, UK; 4 Randall Division of Cell and Molecular Biophysics, and the BHF Centre of Research Excellence, Centre for Biomolecular Spectroscopy, King’s College London, London, UK

**Keywords:** Cardiac energetics, Metabolism, Creatine kinase, Creatine transporter, Transgenic mice

## Abstract

Mice over-expressing the creatine transporter have elevated myocardial creatine levels [Cr] and are protected against ischaemia/reperfusion injury via improved energy reserve. However, mice with very high [Cr] develop cardiac hypertrophy and dysfunction. To investigate these contrasting effects, we applied a non-biased hypothesis-generating approach to quantify global protein and metabolite changes in the LV of mice stratified for [Cr] levels: wildtype, moderately elevated, and high [Cr] (65–85; 100–135; 160–250 nmol/mg protein, respectively). Male mice received an echocardiogram at 7 weeks of age with tissue harvested at 8 weeks. RV was used for [Cr] quantification by HPLC to select LV tissue for subsequent analysis. Two-dimensional difference in-gel electrophoresis identified differentially expressed proteins, which were manually picked and trypsin digested for nano-LC–MS/MS. Principal component analysis (PCA) showed efficient group separation (ANOVA *P* ≤ 0.05) and peptide sequences were identified by mouse database (UniProt 201203) using Mascot. A total of 27 unique proteins were found to be differentially expressed between normal and high [Cr], with proteins showing [Cr]-dependent differential expression, chosen for confirmation, e.g. α-crystallin B, a heat shock protein implicated in cardio-protection and myozenin-2, which could contribute to the hypertrophic phenotype. Nuclear magnetic resonance (¹H-NMR at 700 MHz) identified multiple strong correlations between [Cr] and key cardiac metabolites. For example, positive correlations with α-glucose (*r*² = 0.45; *P* = 0.002), acetyl-carnitine (*r*² = 0.50; *P* = 0.001), glutamine (*r*² = 0.59; *P* = 0.0002); and negative correlations with taurine (*r*² = 0.74; *P* < 0.0001), fumarate (*r*² = 0.45; *P* = 0.003), aspartate (*r*² = 0.59; *P* = 0.0002), alanine (*r*² = 0.66; *P* < 0.0001) and phosphocholine (*r*² = 0.60; *P* = 0.0002). These findings suggest wide-ranging and hitherto unexpected adaptations in substrate utilisation and energy metabolism with a general pattern of impaired energy generating pathways in mice with very high creatine levels.

**Electronic supplementary material:**

The online version of this article (doi:10.1007/s00726-016-2236-x) contains supplementary material, which is available to authorized users.

## Introduction

Increasing myocardial creatine levels, [Cr], by 20–100 %, via over-expression of the plasma membrane creatine transporter (CrT; Slc6A8), protects the murine heart against ischaemia/reperfusion injury and improves functional recovery (Lygate et al. 2012; Whittington et al. 2016).

The underlying mechanisms involve increases in phosphocreatine (PCr), glycogen levels and energy reserve. However, we previously reported that mice with [Cr] twofold higher than wild-type levels (i.e. >140 nmol/mg protein) develop LV hypertrophy (LVH) and chronic heart failure. This reflects the limits on creatine kinase activity to maintain the enlarged creatine pool adequately phosphorylated, thereby limiting the free energy available from ATP hydrolysis (Wallis et al. 2005).

A full understanding of the molecular changes that underpin these adverse effects will be important, if we are to safely exploit the therapeutic potential of moderate creatine elevation. Earlier proteomics analysis using 2D-Difference in-gel electrophoresis (2DIGE) at pI4-10 identified only 7 differentially regulated proteins between hearts from wild-type and CrT over-expressing mice (CrT-OE). Most notably, high [Cr] was associated with lower expression of β-enolase and reduced anaerobic lactate production, suggesting compromised glycolytic capacity (Phillips et al. 2010). However, at the time, there was limited understanding of the dose-related effects of elevating [Cr] and therefore, hearts with moderate and high creatine were analysed as one group, which may have obfuscated the results. The current study addresses this issue by stratifying groups in clearly defined normal, “therapeutic” [Cr] and toxic [Cr] ranges. We have sought to extend our findings using higher resolution 2DIGE proteomics and incorporating an NMR-metabolomics approach for the first time. This has allowed a non-biased exploration of the potential molecular differences underpinning the beneficial versus detrimental cardiac phenotype in transgenic mice with augmented [Cr].

Here, we identify [Cr]-dependent changes in the myocardial proteome with relevance to both cardio-protection and susceptibility to LVH. Elevating creatine to very high levels resulted in wide-ranging effects on metabolic proteins and metabolite levels, which are likely to have a negative impact on the energy providing capacity of the heart.

## Materials and methods

### Chemicals

All chemicals were supplied either by Sigma-Aldrich (Poole, UK), Tocris Bioscience (Bristol, UK), or VWR (Lutterworth, UK).

### Transgenic mouse model and experimental design

We used male mice over-expressing rabbit creatine transporter under control of the MLC2v promoter (CrT-OE) as previously described (Wallis et al. 2005). The Tg55 transgenic line was used, since this line displays the widest range of creatine values, up to fourfold above normal. Transgenic males on a pure C57BL/6J genetic background were mated with C57BL/6J females to produce offspring that are 50 % WT:50 % CrT-OE. Approximately, 17 % of Tg55 mice have LV [Cr] >140 nmol/mg protein. Three groups of mice with creatine levels were pre-defined to ensure a clear [Cr] separation among groups: (a) *Wildtype* (*WT*) *littermates*—LV [Cr] 70—90 nmol/mg protein; (b) *CrT*-*OE medium creatine*—LV [Cr] 110—140 nmol/mg protein; (c) *CrT*-*OE high creatine*—LV [Cr] >160 nmol/mg protein. CrT-OE were backcrossed with C57BL/6J for 10 generations and age-matched wildtype littermates were used as controls.

Mice were kept in specific pathogen-free cages, 12-h light–dark cycle, controlled temperature and humidity, and fed ad libitum with standard chow which is naturally creatine-free (Teklad global 16 % rodent diet) and water ad libitum. Mice were non-fasted at the time of tissue harvest. This investigation was approved by the institutional ethical review committee and conforms to Directive 2010/63/EU of the European Parliament.

#### Echocardiography

At 7 weeks of age, mice were examined by echocardiography to measure LV function and myocardial cross-sectional area. Short axis and Long axis views were obtained under isoflurane anaesthesia using the Visualsonics Vevo 2100. All examinations and measurements were performed by a single operator blinded to mouse genotype and creatine levels. At 8 weeks (1 week post-anaesthetic exposure), mice were killed by cervical dislocation and the heart excised by dissecting LV free from RV, atria and great vessels. After brief washing in heparinised saline and blotting dry, the tissue was freeze-clamped using Wollenberger tongs in liquid nitrogen and then stored at −80 °C, until analysed for total [Cr] (Lygate et al. 2012) using the RV portion and for proteomic and metabolomics analysis using the LV.

#### [Cr] measurements by high-pressure liquid chromatography (HPLC)

Total [Cr] levels were measured by HPLC from homogenised RV samples normalised to non-collagen protein [adapted from (Teerlink et al. 1993)]. We have previously demonstrated that [Cr] is ~7 % lower in the RV, but is highly linearly related to LV levels (ten Hove et al. 2008). A correction factor was, therefore, applied to estimate LV [Cr] from RV [Cr] values based on historical data: LV[Cr] = 1.068(RV[Cr] + 1.9).

#### Proteomics

Previously published methods were followed as in (Yin et al. 2013) and detailed methods are included in the Supplementary Data section.

#### Protein extraction and immunoblotting

LV heart samples were harvested using ice-cold RIPA buffer (Sigma) containing complete protease inhibitor cocktail (Roche) and phosphatase inhibitors as described in (Zervou et al. 2013). Primary antibodies for myozenin-2, total α-crystallin B and phospho-α-crystallin B were purchased from Insight Biotech (Wembley, UK). For normalisation purposes, the myozenin-2 blots were stripped of the primary antibody and re-probed against β-tubulin (Abcam, Cambridge UK). For α-crystallin B, phospho signal was normalised over total and VDAC (Abcam) as a mitochondrial specific protein (Youcef et al. 2015).

#### Catalase assay

Catalase activity was measured in LV tissue lysates using the Amplex Red Catalase Activity Assay Kit (Life technologies) according to the manufacturer’s protocol. Briefly, tissue lysates prepared in RIPA buffer (Sigma) were analysed for protein concentration and the optimal dilution for the assay was determined during optimisation experiments. A standard curve was included in all assays at concentrations of 0–4000 mU/ml. Absorbance was measured at 550 nm against the standard curve, using a Molecular Devices plate reader type spectrophotometer. The catalase activity of each sample was calculated by subtracting the value of the sample from the zero catalase control.

#### qRT-PCR

Total RNA was extracted from LV tissue, using Trizol reagent (Invitrogen) and a phenol/chloroform step before purification by the Qiagen RNeasy Kit (Qiagen) as described before (Zervou et al. 2013). The oligonucleotides used are listed on Suppl. Table 5. For quantification purposes, mRNA levels were normalised over the reference gene 36B4 and using the ΔΔ^*C*t^ method (Livak and Schmittgen 2001).

#### Metabolomics

Extraction of metabolites and ^1^H-NMR are described in Supplementary Data. Correlations between creatine and other metabolites was by Pearson correlation analysis using GraphPad Prism version 5.04.

#### Data analysis

All samples were analysed blinded and randomised to genotype and creatine levels. Data are presented as mean ± SE. Groups were compared by one-way ANOVA unless otherwise stated and differences were considered significant when *P* < 0.05.

## Results and discussion

### [Cr] and hypertrophy in CrT-OE mice

A total of 44 male CrT-OE mice were screened by HPLC measurement of myocardial [Cr]. The three study groups were selected based on pre-defined clearly separated LV [Cr] values, which were estimated from RV creatine measurements (Table 1). LV function was evaluated by echocardiography, 1 week prior to tissue harvest. There were no significant differences between WT and medium [Cr] groups for any parameter. The high [Cr] group had significant LV hypertrophy (myocardial CSA) with mild LV dilatation (end-diastolic area), but preserved contractile function (Fractional area change; Table 1). In support of these changes, [Cr] correlated strongly with myocardial CSA (*r*^2^ = 0.60, *P* = 0.0004). These findings are consistent with previous observations in CrT-OE mice at this age (Phillips et al. 2010).

**Table 1:** Echocardiographic parameters of mice selected for further biochemical analysis based on LV creatine levels estimated from RV measurements

	WT [Cr]	Medium [Cr]	High [Cr]
*n*	10	10	10
LV creatine (nmol/mg protein), mean (range)	81 ± 2 (74–88)	123 ± 2 (113–132)*	220 ± 10 (172–264)^#‡^
Myocardial cross-sectional area (mm^2^)	10.9 ± 0.3	10.9 ± 0.4	12.8 ± 0.5^#‡^
End-diastolic area (mm^2^)	10.2 ± 0.3	10.3 ± 0.4	11.7 ± 0.4^‡^
End-systolic area (mm^2^)	4.6 ± 0.4	4.9 ± 0.32	5.7 ± 0.5
Fractional area change (%)	55 ± 3	53 ± 2	52 ± 3
Heart rate (bpm)	526 ± 8	505 ± 14	535 ± 10

Data are reported as mean ± SEM

*LV* left ventricle

* *P* < 0.05 medium vs WT [Cr], ^#^ *P* < 0.05 high vs medium [Cr], ^‡^ *P* < 0.05 high vs WT [Cr]

### Proteomics

**Fig. 1:**
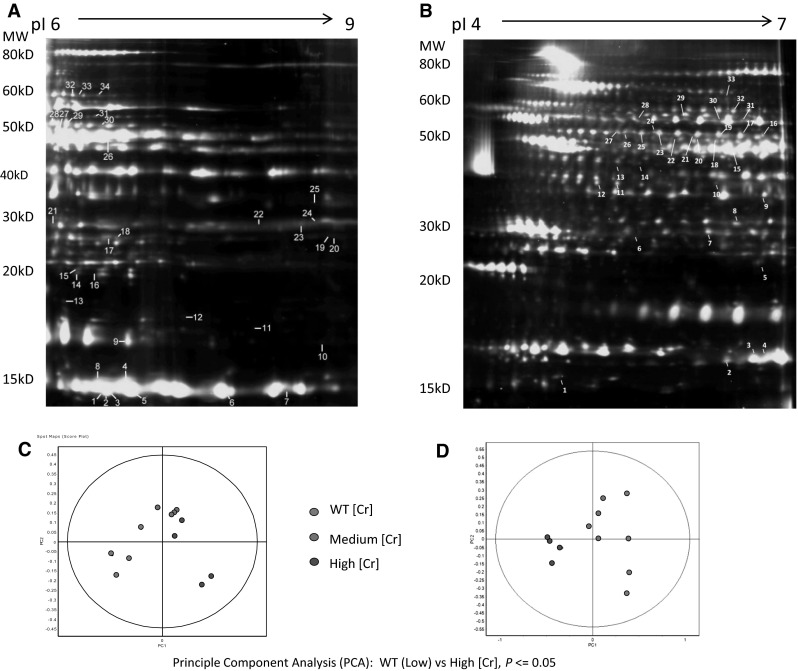
Cardiac protein extracts were analysed by DIGE (*n* = 4 per group). Samples (50 µg) from different groups were labelled with Cy3 or Cy5 and mixture of all samples were used as internal standard and labelled with Cy2. Each pair of Cy3/Cy5 labelled samples were mixed with 50 µg of internal standard and separated by IEF in either pH6-9 (**a**) or pH 4-7 (**b**) IPG strips followed by SDS-PAGE in 12 % large format gels. Fluorescence signals for each dye were scanned using DIGE Imager and spot maps were analysed by DeCyder software. Differentially expressed spots were numbered and peptides identified by LC–MS/MS (complete list of peptides on Supplementary Tables 2 and 3). **c**, **d** Principal component analysis (PCA): WT vs High [Cr], *P* ≤ 0.05. WT group samples (*blue dots*) are well separated from both High [Cr] group (*red dots*) and Medium [Cr] group samples (*green dots*)

A total of 34 differentially regulated peptides were identified in the pI6-9 and 33 in the pI4-7 experiments, respectively (Fig. 1; Suppl Tables 1–3). There is an overlap of four proteins between the two experiments, namely haemoglobin subunit β-1; isocitrate dehydrogenase [NADP] mito; elongation factor Tu, mito; β-enolase. PCA analysis showed efficient separation between groups (*P* ≤ 0.05). Differentially regulated proteins were analysed using a *t* test and the *P* value of <0.05 was set as the statistical significance threshold. These differences per group are listed in Suppl Table 1. We used *n* = 4 samples/group to allow an entire experiment to be run on a single gel, thereby reducing between-experiment variability. This strategy was effective since we were able to confirm changes in β-enolase, glutathione s-transferase and 3-hydroxyacyl-CoA dehydrogenase in response to augmented LV creatine, in agreement with our previous study (Phillips et al. 2010). Novel protein targets that changed dose-dependently with [Cr] were of particular interest and therefore, selected for follow-up.

### Myozenin-2 and Nfatc1 pathway

**Fig. 2:**
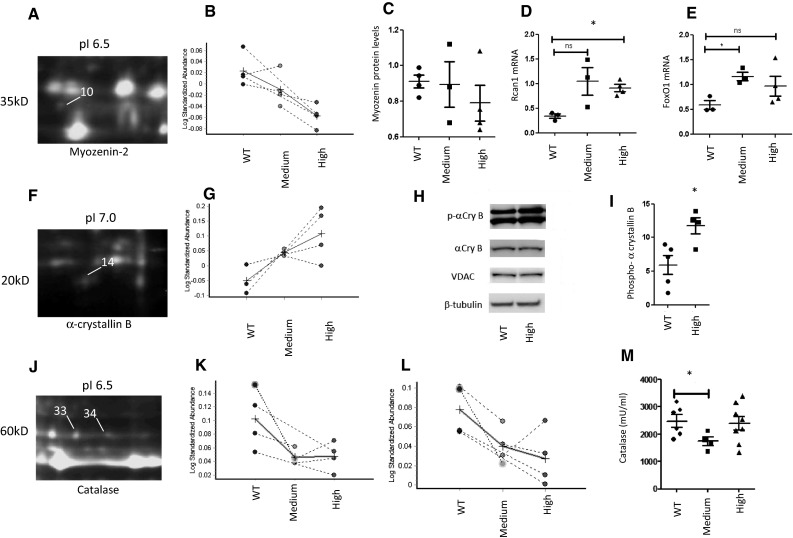
Differentially expressed proteins that changed dose-dependently with LV [Cr]. The left column (**a**, **f**, **j**) shows protein spots enlarged from the full 2-D gels. Myozenin-2 decreased in response to elevated [Cr] in the proteomics data-set (**b**), with a non-significant trend by Western blot (**c**). The regulator of calcineurin, Rcan1, increased as shown by qRT-PCR (D), similarly with FoxO1 (**e**) suggesting downstream activation of the Nfatc1 pathway. α-crystallin B chain increased with [Cr] in both the proteomics data-set (**g**) and by immunoblotting (**h**, **i**). Phospho α-crystallin B expression levels were normalised to total α-crystallin B, VDAC and β-tubulin (**i**; *P* = 0.019). **b**, **g**, **k** and **l** show quantification of proteomics results for myozenin2, α-crystallin B and catalase, respectively. On these graphs, spots in different colours represent WT, Medium and High [creatine] groups *n* = 4 each. On panel J, 33 and 34 indicate the two protein spots that were both identified as catalase. Catalase activity assays showed significant drop between WT and medium but not in high [Cr] group (**m**). Values were normalised over protein

Myozenin-2 is an endogenous calcineurin inhibitor. Reduced myozenin-2 results in unopposed calcineurin activity, relative activation of the nuclear factor of activated T-cells (Nfatc1) pathway and increased susceptibility to LVH (Diedrichs et al. 2004; Frey et al. 2004). Mutations in the myozenin gene are linked to human hypertrophic cardiomyopathy (Ruggiero et al. 2013). Myozenin-2 was down-regulated in the high [Cr] hearts (−21 %; *P* = 0.004 vs. Low; Fig. 2a, b) and this might explain the hypertrophic phenotype. However, this trend was not statistically significant by immunoblotting (Fig. 2c), which used a different set of tissue samples. This may also reflect the differences in sensitivity of the two experimental techniques (proteomics vs immunoblotting). Nevertheless, downstream activation of the Nfatc1 pathway can be inferred by elevated gene expression of regulator of calcineurin (Rcan1) (*P* = 0.017; Fig. 2d) and Foxo1 (Fig. 2e; *P* = 0.01 WT vs medium [Cr] groups). It should be noted that while increased gene expression of Rcan1 is considered a reliable indicator of calcineurin activation, it is not obligatory for myozenin-2 mediated hypertrophy (Ruggiero et al. 2013).

### Alpha crystallin B chain

Alpha crystallin B is a heat shock protein and therefore, plays a role in stabilising proteins under stress conditions. Over-expression protects against necrotic and apoptotic death following ischaemia/reperfusion injury (Ray et al. 2001). Proteomic analysis showed that α-crystallin B chain increases in line with [Cr] (Fig. 2f, g) between WT and medium [Cr] (*P* = 0.0035) or between WT and high [Cr] groups (*P* = 0.0042). Activation of this protein was independently confirmed by immunoblotting for phospho α-crystallin B chain (CryAB) when normalised to total α-crystallin B VDAC and β-tubulin (Fig. 2h, i; *P* = 0.019). This change in α-crystallin B may contribute mechanistically to the protective effects of elevated [Cr] against ischaemia/reperfusion injury (Lygate et al. 2012; Zervou et al. 2016).

### Catalase

The endogenous antioxidant, catalase, was identified by two different peptides using 2DIGE and MS (Fig. 2j–l). In both cases, there was a drop in protein expression in response to augmentation of LV [Cr] and this consistent pattern invited further investigation (ANOVA *P* = 0.047 and *P* = 0.036, respectively for the two peptides). Catalase activity assays using LV lysates from the three groups of study showed a decrease between WT and medium [Cr] groups (*P* = 0.043) (Fig. 2m), but not between medium and high or WT and high [Cr]. A possible reason may be the discrepancy in assay sensitivity levels between proteomics analysis and enzyme activity. Creatine-supplementation has been attributed to direct antioxidant activity (Lawler et al. 2002; Sestili et al. 2006), although this has not been evident in the intact beating heart (Aksentijevic et al. 2014b). One speculative explanation could be that the net antioxidant activity has not changed due to compensatory reduction in catalase.

### Redox regulation

It is notable that approximately half the differentially regulated proteins identified in our experiments are targets for thioredoxin (Fu et al. 2009) (indicated by ^a^ on Suppl. Table 1), which in turn is negatively regulated by thioredoxin interacting protein (Txnip). We have previously reported that Txnip is upregulated in CrT-OE hearts acting as an endogenous inhibitor of further creatine uptake (Zervou et al. 2013).

### Metabolomics

**Fig. 3:**
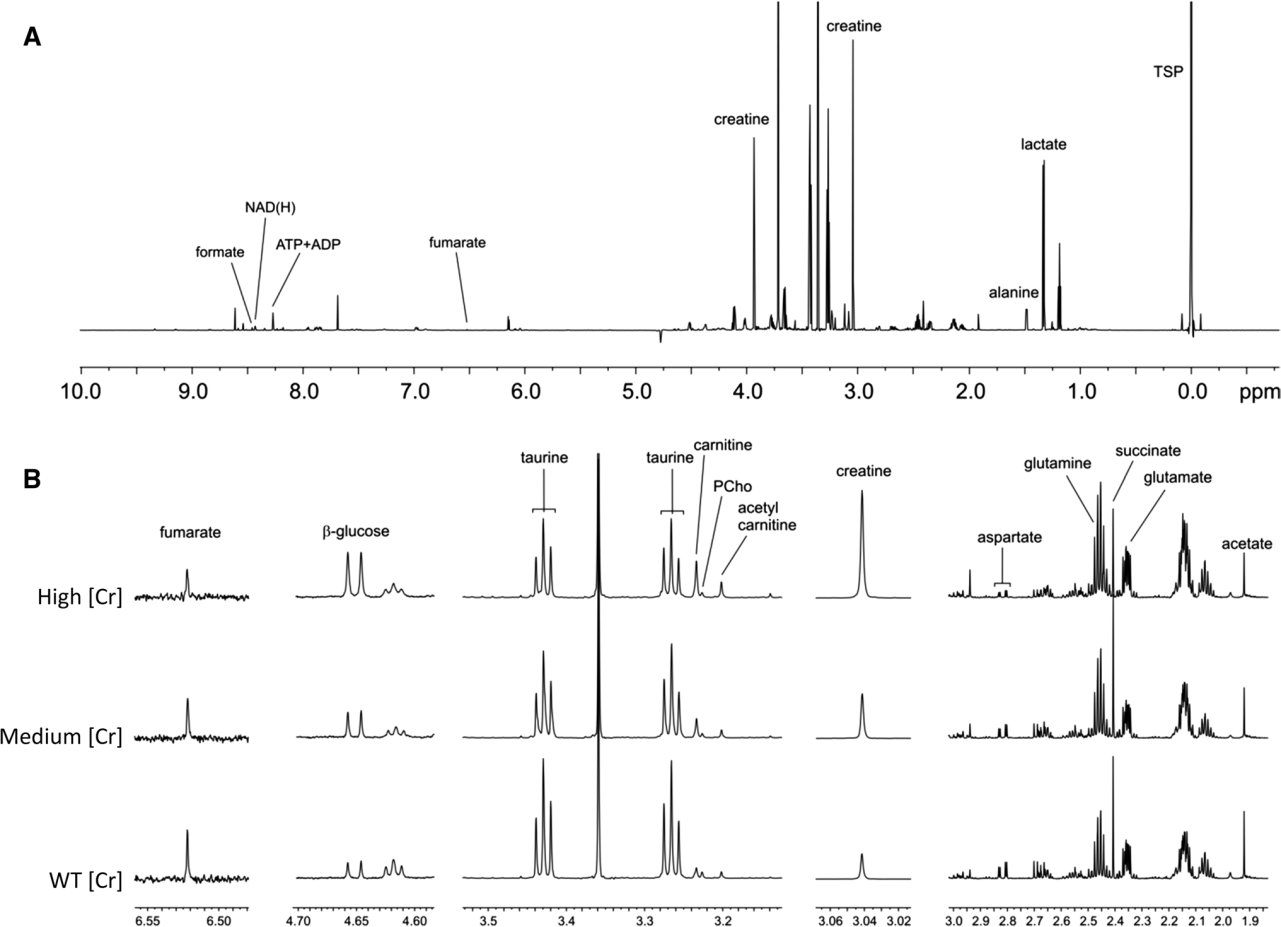
Effects of altered LV [Creatine] on cardiac metabolites. **a** Representative ^1^H-NMR spectrum acquired at 700 MHz. **b** Details from the NMR spectra corresponding to the WT, medium and high creatine groups. Sodium 3-trimethylsilyl-2,2,3,3-tetradeuteropropionate (TSP) was added to the samples for chemical shift calibration and peak quantification. N.B. There are two peaks for taurine, each representing a different methylene group (Mayr et al. 2009)

Initial partial least squares discriminant analysis (PLS-DA) of the ^1^H-NMR results showed a good separation of the three groups for the aqueous metabolites (*n* = 6 each) (Suppl Fig. 1A). Representative NMR spectra are shown in Fig. 3.

**Fig. 4:**
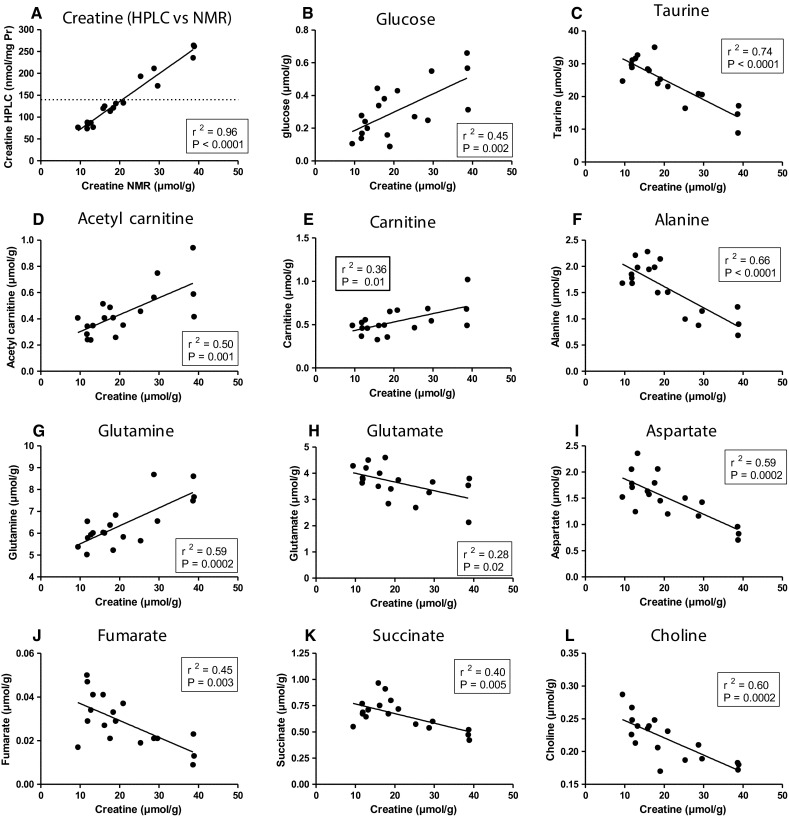
Metabolomic analysis of selected aqueous and lipid metabolites shown with respect to LV creatine levels measured by NMR (based on data listed on Table 2 and Suppl Table 4). There are *n* = 6 samples from each of the three groups to provide a continuum of creatine values ranging from 9 to 13 µmol/g for wildtype, 15–21 µmol/g for medium [Cr] and 25–39 µmol/g for high [Cr]

*Aqueous metabolites* All detected metabolites are shown in Table 2 and corresponding correlations with LV [Cr] levels in Fig. 4. Creatine detected by HPLC and used for group stratification strongly correlated to the values obtained by ^1^H-NMR (Fig. 4a; *P* < 0.0001), providing independent validation for accuracy and reproducibility of estimating LV [Cr] from RV [Cr] measurements. Unexpectedly, we observed multiple strong correlations between metabolite levels and [Cr], for example, a [Cr]-dependent increase in glucose levels (*P* = 0.002; Fig. 4b) and accumulation of acetyl-carnitine (*P* = 0.001; Fig. 4d) and carnitine (*P* = 0.01; Fig. 4e), which suggests an abundance of mitochondrial acetyl-CoA levels (Longnus et al. 2001). In contrast, there was no change in myocardial triglyceride (Suppl Table 4) or lactate levels (Table 2) with elevated [Cr]. The Krebs cycle intermediates, fumarate and succinate, were both reduced with high [Cr] (Fig. 4j, k) and there were strong effects on metabolites associated with anaplerotic flux into the Krebs cycle, e.g. reduced levels of glutamate (Fig. 4h), alanine (Fig. 4f) and aspartate (Fig. 4i). It seems likely that the strong negative correlation with taurine (Fig. 4c) functions to balance the osmotic effect of creatine accumulation, since both are abundant osmolytes (Ito et al. 2008). Phosphocholine levels were reduced by 23 % in the high [Cr] hearts (Suppl Table 4), which could reflect the burden of increased creatine biosynthesis, since choline is a methyl donor for the co-factor s-adenosyl methionine. However, it has been estimated that 95 % of total [Cr] is located in skeletal muscle (Persky and Brazeau 2001), so even a fourfold [Cr] elevation in the heart will have minimal impact on whole body demands.

**Table 2:** Aqueous metabolites as detected and quantitated by ^1^H-NMR

Metabolite	WT (*n* = 6)	SEM	Medium (*n* = 6)	SEM	High (*n* = 6)	SEM	One-way ANOVA		
							WT/M	M/H	WT/H
Formate	10.006	9.749	17.687	11.207	9.537	6.062	ns	ns	ns
NAD(H)	0.517	0.022	0.602	0.067	0.609	0.036	ns	ns	ns
ATP + ADP	2.139	0.056	2.323	0.186	2.141	0.122	ns	ns	ns
Fumarate	0.036	0.005	0.031	0.003	0.018	0.002	ns	ns	**
Glucose	0.188	0.026	0.306	0.061	0.434	0.073	ns	ns	*
Creatine (CH_2_)	11.811	0.535	17.984	0.773	33.322	2.500	*	***	***
Glycine	0.539	0.024	0.519	0.027	0.505	0.022	ns	ns	ns
Taurine	29.766	1.159	27.257	1.775	16.413	1.799	ns	**	***
Carnitine	0.476	0.027	0.498	0.058	0.647	0.084	ns	ns	ns
Phosphocholine	0.246	0.011	0.222	0.012	0.187	0.005	ns	ns	**
Acetyl-carnitine	0.310	0.027	0.405	0.038	0.620	0.080	ns	*	**
Creatine (CH_3_)	10.067	0.483	15.619	0.654	28.861	2.170	*	***	***
Aspartate	1.778	0.160	1.619	0.120	1.096	0.132	ns	*	*
Glutamine	5.780	0.215	6.051	0.218	7.443	0.481	ns	*	**
Succinate	0.672	0.030	0.804	0.047	0.521	0.027	ns	***	*
Glutamate	4.039	0.139	3.685	0.243	3.183	0.265	ns	ns	ns
Acetate	0.438	0.049	0.396	0.061	0.386	0.028	ns	ns	ns
Alanine	1.862	0.084	1.892	0.133	0.971	0.080	ns	***	***
Lactate	10.499	0.550	10.709	0.532	11.223	0.873	ns	ns	ns

Data from the three groups were analysed by one-way ANOVA and Bonferroni multiple comparisons post hoc test using Graphpad Prism. All concentrations are calculated with respect to TSP as a reference standard and normalised to tissue wet weight. Concentrations are given in μmol/g wet weight

*Ns* non-significant difference

* *P* ≤ 0.05; ** *P* ≤ 0.01; *** *P* ≤ 0.001

The increased levels of glutamine we observed could also theoretically contribute to anaplerotic flux via glutamate and α-ketoglutarate, however, this was not detected in the working rat heart perfused with ^13^C-labelled glutamine, even under pro-anaplerotic conditions (Lauzier et al. 2013). Instead, the authors detected changes in lipid metabolism that could be blocked by inhibiting the hexosamine biosynthetic pathway (HBP) (Lauzier et al. 2013). Glutamine is required for the first step in the HBP which leads to protein O-GlcNAcylation and thereby influences a multitude of cellular functions including known metabolic and cardioprotective proteins (Bond and Hanover 2015). Perfusion with glutamine has been shown to protect against ischaemia/reperfusion injury and again this could by blocked by addition of an HBP inhibitor (Liu et al. 2007). Further work is merited to test the hypothesis that elevated glutamine levels contribute to ischaemic protection in the CrT-OE mice via activation of the HBP.

*Lipid metabolites* Initial analysis by supervised PLS-DA showed a weak separation of the three groups (Suppl Fig. 1B). The full list of lipid metabolites as quantitated by ^1^H NMR, is shown on Suppl. Table 4. Some choline is apparently incorporated into additional phosphatidylcholine, which was elevated with high [Cr], while sphingolipid was reduced. Exogenous phosphocreatine has previously been shown to interact with phospholipids and stabilise the plasma membrane (Tokarska-Schlattner et al. 2012) and it is possible that this may also subtly alter the biochemical composition. Other lipid metabolites were not altered to a physiologically relevant level with the caveat that ^1^H-NMR has low sensitivity (Hinterwirth et al. 2014).

### Integrating proteomics and metabolomics

**Fig. 5:**
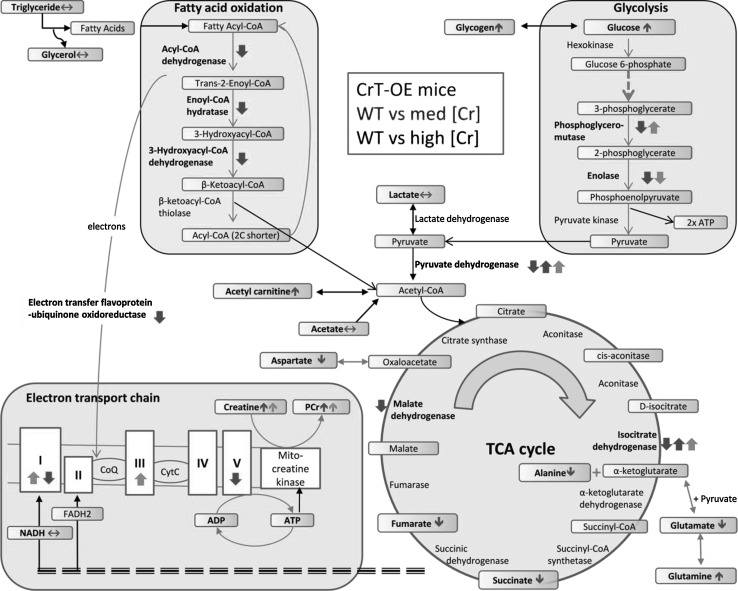
Schematic integrating the proteomic and metabolomic changes resulting from elevating myocardial creatine in vivo. *Colour-coded arrows* indicate the directional change of significantly altered proteins and metabolites in wild-type (WT) versus creatine transporter over-expressing mice (CrT-OE): *green arrows* represent mice with medium [Cr] levels and red arrows mice with high [Cr]. The two *red up/down arrows* correspond to cases when two subunits of the same molecule changed in the opposite direction. *Horizontal arrows* correspond to metabolites that were identified, but did not change significantly. Very high [Cr] had detrimental effects on multiple energy-generating pathways. *TCA* tricarboxylic acid cycle, *PCr* phosphocreatine, *NADH* nicotinamide adenine dinucleotide, *FADH*_*2*_ flavin adenine dinucleotide

Many of the differentially regulated proteins are involved in energy metabolism and we have attempted to integrate all the relevant proteomic and metabolomic data in a single diagram (Fig. 5). Although we observe clear [Cr] dose-dependency for many metabolites, indicating a continuum, significant changes are only observed in the high [Cr] group. Likewise, we observed reduced expression of multiple metabolic enzymes, but predominantly in the high [Cr] group. Thus, a general pattern emerges of impaired energy-generating pathways in mice with very high [Cr], as follows:

*Glucose metabolism* impaired glycolysis is in agreement with our previous study, which showed that reduced enolase expression impacted on capacity for lactate production, but only in mice with [Cr] >140 nmol/g protein (Phillips et al. 2010). Elevated PCr may reduce the need for glycolysis to power short-term increases in energy requirements (Safdar et al. 2008). It is notable that total glucose levels are elevated which may arise from either the intra or extracellular pool, suggesting that there is reduced glucose utilisation, consistent with our previous study. Excess glucose is probably converted to glycogen and we have previously shown that glycogen levels are positively correlated with myocardial [Cr] (Lygate et al. 2012). It is not possible to infer the impact (if any) on glucose oxidation since we observed changes in pyruvate dehydrogenase (PDH) subunits that are directionally opposed and lactate was unchanged. Ideally, we would have measured PDH activity biochemically or by hyperpolarised ^13^C-pyruvate, however, the former requires an entire mouse heart and the latter would require pre-stratification for [Cr] using in vivo ^1^H-MRS making it impractical and prohibitively expensive. As a surrogate measure, we quantified PDK4 mRNA, since this is a major regulator of PDH activity in the heart (Sugden and Holness 2006) and observed no differences in expression (WT 1.8 ± 0.38; medium 2.317 ± 0.23; high 1.8 ± 0.34, respectively). GLUT1-OE mice also have increased glucose uptake and glycogen stores and are protected against pressure-overload heart failure (Liao et al. 2002) and show improved tolerance to ischaemia (Luptak et al. 2007).

*Fatty acid oxidation* (*FAO*) the expression of three key enzymes involved in β-oxidation were reduced in high [Cr] compared to WT hearts (but not in moderate [Cr]), strongly suggesting a deficit in the ability to utilise fatty acids. Presumably, there is commensurate reduction in fatty acid uptake since we do not observe accumulation of lipids, with a trend for reduced triglycerides and significantly lower sphingolipids in the high [Cr] group.

*Tricarboxylic acid* (*TCA*) *cycl*e Our findings also suggest an imbalance in the TCA cycle with potential restrictions at the level of isocitrate dehydrogenase and malate dehydrogenase. Changes in metabolite levels suggest altered anaplerotic flux, which might represent a response to these bottlenecks (e.g. glutamate feeding into α-ketoglutarate; aspartate conversion to oxaloacetate). It is surprising that acetyl-carnitine is elevated in high [Cr] hearts since this indicates that substrate availability is higher than demand. Excess acetyl-CoA is converted to acetyl-carnitine and exported out of the mitochondria, to be broken down to its constituent parts in the cytosol, where it may inhibit fatty acid uptake (Longnus et al. 2001). Overall, this suggests that despite acetyl-CoA production via glycolysis and FAO likely to be reduced in the high [Cr] hearts, the rate of acetyl-CoA production still outstrips the capacity of the TCA cycle.

Our analysis represents a snap-shot of protein expression and metabolite concentrations and may not represent dynamic flux through the system. It would have been informative to quantify substrate preference, e.g. by radiolabeled uptake experiments or ^13^C-hyperpolarisation studies. However, male mice with very high [Cr] are rare (~8 % of all offspring from heterozygote mating) and it took us several years to breed sufficient mice for the current study. Thus, complex experiments requiring high animal numbers are not practicable.

Theoretically, we might have gained further insights using computer modelling. However, the fact that we observed such large and widespread changes in metabolite concentrations is a truly surprising result, particularly given that creatine is at the terminus of energy-generating pathways with few known feedback mechanisms. Hence, existing in silico models are of limited utility in understanding these findings, which suggest deep interconnections that are not part of current metabolic models. For example, the CardioNet metabolic flux model we have used previously (Aksentijevic et al. 2014a) is insensitive to altered creatine levels.

A further potential limitation is that [Cr] correlates closely with LV hypertrophy (Wallis et al. 2005), which has its own metabolic sequelae, raising the possibility that the correlations observed between [Cr] and other metabolites are simply an epiphenomenon. The metabolic response to LVH is characterised by an increase in glycolytic and anaplerotic flux, manifesting as elevated lactate, alanine and aspartate in aortic banded mice and rats (Kolwicz et al. 2012; Sorokina et al. 2007). A similar pattern was observed in hyperthyroid-induced LVH where glucose and glycogen levels were also reduced (Atherton et al. 2011). All these responses are directionally opposite to what we observe in high [Cr] hearts suggesting that the presence of LVH is not driving the metabolic phenotype. It should also be noted that the severity of LVH in our study is relatively mild. Myocardial cross-sectional area increased by 17 % in high vs normal [Cr], compared with an increase of 56 % in the transverse aortic constriction model (Lygate et al. 2007), therefore, the driving force for metabolic remodelling is not as strong.

Our model of augmented cardiac creatine is a result of transgenesis and therefore, a ‘forced’ metabolic phenotype that does not occur naturally. Whether there is metabolic feedback at physiological creatine levels that is lost under the pathologically low [Cr] levels observed in the failing heart remains to be established. Nevertheless, our findings are highly informative when considering target levels for therapeutic [Cr] aimed at improving cardiac energetics, most likely via pharmacological activation of the creatine transporter (Zervou et al. 2016). Only the high [Cr] group had a severe metabolic phenotype, which supports the concept of a safe window for creatine elevation between 20 and 100 % above wild-type levels (i.e. corresponding to the medium [Cr] group), which we previously demonstrated does not impact on in vivo function and protects against ischaemia–reperfusion injury (Lygate et al. 2012).

Finally, we took a non-biased approach to identify proteomic and metabolic adaptations in response to elevated myocardial creatine levels in vivo. In mice with very high [Cr], we observed reduced expression in multiple proteins involved in energy generation, implying impairment of glycolysis, fatty acid oxidation and the TCA cycle, resulting in a substrate rich, but energy-poor heart. Surprisingly, strong correlations were observed between creatine tissue levels and many key metabolites suggesting the existence of hitherto unsuspected feedback mechanisms. The potential link between creatine and glucose uptake is of particular interest for future study.

## Electronic supplementary material

Below is the link to the electronic supplementary material.

Supplementary material 1 (DOCX 244 kb)

## Abbreviations

DIGE: Difference in-gel electrophoresis
^1^H-NMR: Proton nuclear magnetic resonance spectroscopy
LC–MS/MS: Liquid chromatography tandem mass spectroscopy
PCA: Principal component analysis
Cr: Creatine
CrT: Creatine Transporter
LVH: Left ventricular hypertrophy
PCr: Phosphocreatine
