# Supplementary material for: Proteomic and metabolomic changes driven by elevating myocardial creatine suggest novel metabolic feedback mechanisms
Source: Amino Acids. 2016 May 3;48:1969–81. doi: 10.1007/s00726-016-2236-x (PMC4974297; doi:10.1007/s00726-016-2236-x)
Supplement: Supplementary file 1 — Supplementary material 1 (DOCX 244 kb) [file 726_2016_2236_MOESM1_ESM.docx]

Data Supplement

**HPLC.** An isocratic mobile phase (0.215M KH_2_PO_4_, 0.23mM C_16_H_37_NO_4_S and 3.5% v/v acetonitrile (10754361) in water was used in our analysis. Both Standards and samples were thawed and kept on ice throughout before injecting 100μl in the system following equilibration by running mobile phase through the column at 0.7ml/min for 20 mins. The same flow was maintained throughout experiments. Detection was performed at 206nm and total time between injections was 22 mins. All compounds (Cr, PCr, AMP, ADP, ATP) were quantified by measurement of peak area.

**Proteomics.** Tissues were homogenized in DIGE lysis buffer (8M urea, 4% w/v CHAPS, 30mM Tris-Cl, pH 8.5) and soluble proteins underwent DIGE labelling (GE Healthcare). Fluorescent dyes were added to the protein at dye/protein ratio of 200pmol/50μg for 30 min on ice. The reaction was then stopped by scavenging non-bound dyes with 10mM L-lysine (Sigma). Before two-dimensional gel electrophoresis samples were mixed with 2x buffer (8M urea, 4% w/v CHAPS, 2% w/v DTT, 2% v/v Pharmalytes), diluted in rehydration solution (8M urea, 0.5% w/v CHAPS, 0.2% w/v DTT, 0.2% v/v Pharmalytes) and loaded on IPG strips (18cm, pH 4-7 and pH 6-9, nonlinear, GE healthcare). Strips were focused at 0.05 mA/IPG strip for 42 kVh (for pH 4-7 strips) or 45kVh (for pH 6-9 strips) at 20^o^C after overnight rehydration. IEF strips were equilibrated with equilibration buffer (6M urea, 2% w/v SDS, 30% v/v glycerol, 50mM Tris, pH=8.8) plus 10mg/mL DTT for 15min then replaced with equilibration buffer plus 48mg/mL IAA. The strips were transferred on top of large format 12% Tris-Glycine polyacrylamide gels (12% total acrylamide, 2.6% cross-linking) and SDS-PAGE was performed using 17W/gel until the dye-front migrate off the gels. The protein gel incorporates a pH gradient, and each protein moves only until it reaches its isoelectric point (pI).  The pI is the pH where a protein has no net charge, meaning that it stops migrating, focusing tightly into a protein spot within 0.01 pH unit of its pI. Two independent experiments were run at pI 6-9 and pI4-7 (the latter to increase resolution). Fluorescent images were acquired using an Ettan DIGE imager (GE healthcare) and gels were silver-stained using Plus One silver staining kit (GE healthcare) so that differentially expressed proteins could be located and excised. Fluorescent images were analysed by DeCyder software (version 7.0, GE healthcare) and protein spots with fold change > 1.2 or < -1.2 and p<0.05 were manually picked. Picked spots were subjected to overnight in-gel tryptic digestion using a robot (ProGest, Digilab). After tryptic digestion the peptide mixture was resuspended in 20μl of 2% ACN, 0.05% TFA in HPLC H_2_O, then separated by reversed phase nano-flow HPLC (PepMap C18, 25cm x 75um, Dionex RSLC) (0-15min, 2%B-35%B; 15-20min, 99%B; 20-30min, 2%B; A=0.1% FA in HPLC H_2_O; B=80% ACN, 0.1% FA in HPLC H_2_O) and directly analysed by LTQ Orbitrap XL using full scan mode m/z range 400-1600 with Orbitrap, resolution 60000 (at m/z 400), lock mass m/z = 445.1200. MS/MS was performed using CID in ion trap on the top 3 ions with dynamic exclusion.

Raw files were searched against UniProt/SwissProt mouse database (version 2012_03), using Mascot 2.3.01. The mass tolerance was set at 10 ppm for the precursor ions and at 0.8 Da for fragment ions. Carboxyamidomethylation of cysteine was used as a fixed modification and oxidation of methionine as variable modification. Two missed cleavages were allowed.

Search results were loaded into Scaffold (version 4.3.0) and the following filters were used: peptides probability > 95%, protein probability >99%, minimum 2 unique peptides identified.

**Dual-phase Extraction of Metabolites from Heart Tissue.** Heart weights were recorded prior to extraction. The heart was powdered frozen and the powder was vortexed thoroughly with 2ml each of ice-cold methanol, chloroform and ultra-purified, distilled water. The mixture was then centrifuged for 1 hour at 3600 rpm at 4°C to achieve separation into an upper aqueous phase, a middle protein phase and a lower chloroform phase. The aqueous layer was transferred into a new 15 ml polyethylene tube and combined with 20-30 mg chelex, followed by vortexing and centrifugation at 3600rpm for 5 minutes at 4°C. The aqueous supernatant was added to a new 15 ml polyethylene tube and mixed with 10-20µl universal indicator solution, followed by freeze-drying and then storage at -80°C. The lipid layer was removed from the original tube (being careful not to disturb the protein layer), transferred into a glass scintillation vial (loosely covered with parafilm wax) and left over night in a fume cupboard to evaporate under ambient laboratory conditions.

**^1^H NMR analysis of dual-phase-extracted metabolite samples.** Dual-phase-extracted mouse heart metabolite samples were analysed using a vertical-bore, ultra-shielded Bruker 16.4 T (700 MHz) spectrometer with a cryoprobe at 298K. Freeze-dried aqueous extracts were re-dissolved in 600µL deuterated water (D_2_O containing 8 g/L NaCl, 0.2 g/L KCl, 1.15 g/L Na_2_HPO_4_, 0.2 g/L KH_2_PO_4_ and 0.0075% w/v trimethylsilyl propanoic acid) was added, vortex mixed and the pH was adjusted to 7 using 1M HCl or 1M NaOH where necessary. Dried lipid extracts were reconstituted in 600µL deuterated chloroform (CDCl_3_) containing 0.05% v/v tetramethylsilane. For aqueous samples, a NOESY 1D pulse sequence was used, with 64 scans, 4 dummy scans and 20.5 ppm sweep width, 4s pre-scan delay, 90° flip angle and experiment duration of 9.1 minutes. For lipid samples, a ZG30 pulse sequence was used, with 64 scans, 4 dummy scans 16.1 ppm sweep width, 5s pre-scan delay, 30° flip angle and experiment duration of 11.5 minutes. TopSpin (version 2.1) software was used for data acquisition and for metabolite quantification.  Assignment of metabolites to their respective peaks was carried out based on previously obtained in-house data and confirmed by chemical shift. Peak areas were normalised to the TSP or TMS peaks and metabolite concentrations quantified per gram tissue.

| **Experiment** | **No** | **T-test** | **Av. Ratio** | **Protein name** | **UniProt ID** | **Function** |
| --- | --- | --- | --- | --- | --- | --- |
|  |  |  |  | ***WT vs Medium*** |  |  |
| **pI 6-9** | 1 | 0.048 | 1.2 | ^a^Nadh dehydrogenase iron-sulfur prot 6 mito | NDUS6_MOUSE | Mito resp chain complex 1 |
|  | 8 | 0.049 | 1.48 | Cytochrome b-C1 complex | QCR7_MOUSE | ETC |
|  | 10 | 0.0095 | 1.62 | Myoglobin | MYG_MOUSE | Response to peroxide |
|  | 14 | 0.0035 | 1.26 | α crystallin B chain | CRYAB_MOUSE | Redox regulation |
|  | 17 | 0.014 | -1.28 | Flavin reductase (NADPH) | BLVRB_MOUSE | Biliverdin reductase activity |
|  | 18 | 0.018 | -1.21 | Glutathione S-transferase Mu1 | GSTM1_MOUSE | Susceptibility to toxicity |
|  | 21 | 0.028 | 1.24 | Phosphoglycerate mutase 2 | PGAM2_MOUSE | Glycolysis |
|  | 21 | 0.028 | 1.24 | Enoyl-CoA delta isomerase 1, mito | ECI1_MOUSE | Fatty acid, lipid metabolism |
|  | 21 | 0.028 | 1.24 | Coiled-coil-helix domain-cont protein 3, mito | CHCH3_MOUSE | Imm organization |
|  | 27 | 0.019 | 1.2 | ^a^Isocitrate dehydrogenase [NADP], mito | IDHP_MOUSE | 2-oxoglutarate metabolism |
|  | 27 | 0.019 | 1.2 | Elongation factor Tu, mitochondrial | EFTU_MOUSE | GTP catabolic process |
|  | 28 | 0.037 | -1.24 | Beta-enolase | ENOB_MOUSE | Glycolysis |
| **pI4-7** | 4 | 0.0061 | 1.3 | ^a^NADH Dehydrogenase [ubiq] 1 a | NDUA5_MOUSE | ETC |
|  | 5 | 0.033 | 1.24 | α crystallin B chain | CRYAB_MOUSE | Redox regulation |
|  | 18 | 0.0023 | 1.36 | ^a^Isocitrate dehydrogenase [NADP] mito | IDHP_MOUSE | 2-oxoglutarate metabolism |
|  | 18 | 0.0023 | 1.36 | Elongation factor Tu, mitochondrial | EFTU_MOUSE | Protein translation |
|  | 18 | 0.0023 | 1.36 | ^a^Pyruvate dehydrogenase E1 compl. s.u. α | IDHP_MOUSE | pyruvate metabolism |
|  | 18 | 0.0023 | 1.36 | ^a^Isocitrate dehydrogenase [NADP] mito | IDHP_MOUSE | 2-oxoglutarate metabolism |
|  |  |  |  | ***Medium vs High*** |  |  |
| **pI 6-9** | 11 | 0.023 | -1.22 | ^a^Nucleoside diphosphate kinase B | NDKB_MOUSE | Regulates contractility |
|  | 12 | 0.024 | -1.39 | Unidentified | - | - |
|  | 16 | 0.015 | 1.52 | Transgelin-2 | TAGL2_MOUSE | Cancer |
|  | 28 | 0.0075 | -1.23 | Beta-enolase | ENOB_MOUSE | Glycolysis |
|  | 26 | 0.043 | -1.27 | ^a^Isocitrate dehydrogenase [NADP] mito | IDHP_MOUSE | Mitochondrial redox |
| **pI 4-7** | 6 | 0.015 | 1.50 | Myosin-6 | MYH6_MOUSE | Protein binding |
|  | 19 | 0.022 | -1.31 | Beta-enolase | ENOB_MOUSE | Glycolysis |
|  | 20 | 0.007 | -1.35 | Beta-enolase | ENOB_MOUSE | Glycolysis |
|  | 22 | 0.0019 | -1.37 | Beta-enolase | ENOB_MOUSE | Glycolysis |
|  | 23 | 0.0035 | -1.41 | Beta-enolase | ENOB_MOUSE | Glycolysis |
|  | 25 | 0.0038 | -1.47 | Beta-enolase | ENOB_MOUSE | Glycolysis |
|  | 26 | 0.012 | -1.46 | Beta-enolase | ENOB_MOUSE | Glycolysis |
|  | 29 | 0.042 | 1.44 | ^a^ATP synthase s.u. α mito | ATPA_MOUSE | ATP synthesis |
|  | 31 | 0.038 | 1.22 | UTP-glucose-1-phosphate uridylyltransferase | UGPA_MOUSE | Glycogenesis |
|  | 31 | 0.038 | 1.22 | ^a^ATP synthase s.u. α mito | ATPA_MOUSE | ATP synthesis |
|  |  |  |  | ***WT vs High*** |  |  |
| **pI 6-9** | 9 | 0.036 | -1.48 | Myoglobin | MYG_MOUSE | Response to peroxide |
|  | 13 | 0.037 | -1.21 | ^a^Nucleoside diphosphate kinase B | NDKB_MOUSE | ATP binding |
|  | 14 | 0.044 | 1.74 | α crystallin B chain | CRYAB_MOUSE | Anti-apoptotic, Redox |
|  | 15 | 0.042 | 1.35 | α crystallin B chain | CRYAB_MOUSE | Anti-apoptotic, Redox |
|  | 16 | 0.0052 | 1.51 | Transgelin-2 | TAGL2_MOUSE | Differentiation marker |
|  | 19 | 0.0021 | -1.3 | 3-hydroxyl-CoA-dehydrogenase type-2 | HCD2_MOUSE | Fatty acid oxidation |
|  | 20 | 0.0024 | -1.2 | Protein NipSnap homolog 2 | NIPS2_MOUSE | Plasma membrane |
|  | 23 | 0.023 | -1.3 | Phosphoglycerate mutase 2 | PGAM2_MOUSE | Glycolysis |
|  | 25 | 0.042 | -1.33 | ^a^ATP synthase subunit gamma, mito | ATPG_MOUSE | ATP synthesis |
|  | 25 | 0.042 | -1.33 | ^a^PDZ and LIM domain protein 5 | PDLI5_MOUSE | Hypertrophy |
|  | 28 | 0.0005 | -1.53 | Beta-enolase | ENOB_MOUSE | Glycolysis |
|  | 31 | 0.013 | -1.34 | ^a^NADH dehydrogenase | NDUV1_MOUSE | ETC |
|  | 31 | 0.013 | -1.34 | ^a^Isocitrate dehydrogenase [NADP] mito | IDHP_MOUSE | 2-oxoglutarate metabolism |
| **pI 4-7** | 1 | 0.0065 | 1.22 | 10kDa heat shock protein, mitochondrial | CH10_MOUSE | Protein folding |
|  | 4 | 0.048 | 1.41 | ^a^NADH Dehydrogenase [ubiq] 1 a | NDUA5_MOUSE | ETC |
|  | 5 | 0.043 | 1.46 | α crystallin B chain | CRYAB_MOUSE | Redox, anti-apoptotic |
|  | 8 | 0.00076 | -1.30 | ^a^Enoyl-CoA hydratase, mitochondrial | ECHM_MOUSE | Fatty acid beta oxidation |
|  | 9 | 0.0061 | -1.23 | Hydroxyacyl-Coenzyme A dehydrogenase, mito | HCDH_MOUSE | Fatty acid beta oxidation |
|  | 10 | 0.0041 | -1.21 | Myozenin-2 | MYOZ2_MOUSE | Susceptibility to hypertrophy |
|  | 10 | 0.0041 | -1.21 | ^a^Four and a half LIM domains protein 2 | FHL2_MOUSE | anti-apoptotic |
|  | 11 | 0.014 | -1.32 | ^a^Malate dehydrogenase | MDHC_MOUSE | TCA cycle |
|  | 12 | 0.012 | -1.31 | ^a^Pyruvate dehydrogenase E1 compl. s.u. α | ODPA_MOUSE | Glycolysis |
|  | 12 | 0.012 | -1.31 | ^a^Malate dehydrogenase |  | Carbohydrate metabolism |
|  | 13 | 0.0018 | -1.27 | ^a^Isocitrate dehydrogenase [NAD] su alpha mito | IDH3A_MOUSE | TCA cycle |
|  | 14 | 0.026 | -1.29 | ^a^Isocitrate dehydrogenase [NAD] su alpha mito | IDH3A_MOUSE | TCA cycle |
|  | 16 | 0.035 | -1.41 | Beta-enolase | ENOB_MOUSE | Glycolysis |
|  | 17 | 0.036 | -1.40 | Beta-enolase | ENOB_MOUSE | Glycolysis |
|  | 18 | 0.0071 | 1.35 | Elongation factor Tu, mitochondrial | EFTU_MOUSE | GTP catabolic process |
|  | 18 | 0.0071 | 1.35 | ^a^Pyruvate dehydrogenase E1 compl. s.u. α | IDHP_MOUSE | 2-oxoglutarate metabolism |
|  | 18 | 0.0071 | 1.35 | ^a^Isocitrate dehydrogenase [NADP] mito | IDH3A_MOUSE | TCA cycle |
|  | 19 | 0.01 | -1.61 | Beta-enolase | ENOB_MOUSE | Glycolysis |
|  | 20 | 0.0052 | -1.58 | Beta-enolase | ENOB_MOUSE | Glycolysis |
|  | 21 | 0.0037 | -1.27 | Beta-enolase | ENOB_MOUSE | Glycolysis |
|  | 22 | 0.00048 | -1.59 | Beta-enolase | ENOB_MOUSE | Glycolysis |
|  | 23 | 0.0015 | -1.48 | Beta-enolase | ENOB_MOUSE | Glycolysis |
|  | 24 | 0.0036 | -1.26 | Alpha-enolase | ENOA_MOUSE | Metabolism-cancer |
|  | 25 | 0.0019 | -1.47 | Beta-enolase | ENOB_MOUSE | Glycolysis |
|  | 26 | 0.035 | -1.37 | Beta-enolase | ENOB_MOUSE | Glycolysis |
|  | 33 | 0.002 | -1.24 | Very long-chain specific acyl-CoA dehydrogenase, nitochondrial | ACADV_MOUSE | Fatty acid beta oxidation |
|  | 33 | 0.002 | -1.24 | Electron transfer flavoprotein-ubiquinone oxidoreductase, mitochondrial | ETFD_MOUSE | ETC |
|  | 33 | 0.002 | -1.24 | Prelamin-A/C | LMNA_MOUSE | Cardiomyocyte senescence |

Supplementary Table 1. Differentially regulated protein spots following 2DIGE and LC-MS-MS analysis from two independent experiments, at pI 6-9 and pI 4-7 respectively. Results are collectively presented from both experiments. Picklists were generated using the following parameters: t-Test p<0.05 and fold>1.2 or <-1.2 for each comparison, and ANOVA<0.05 for all 3 groups. Spots were manually picked and digested with trypsin, then analyzed by nano-LC-MS/MS. Results were searched against mouse database (UniProt 201203) using Mascot. ^a^ denotes proteins known to be targets of thioredoxin.

| No. | Protein name | Protein accession numbers | Protein molecular weight [2] | Protein identification probability | No. of unique peptides | No. of unique spectra | No. of total spectra | Sequence coverage |
| --- | --- | --- | --- | --- | --- | --- | --- | --- |
| 1 | NADH dehydrog. [ubiquin] iron-sulfur prot 6, mito | NDUS6_MOUSE | 13,019.60 | 98.50% | 1 | 1 | 2 | 12.90% |
| 2 | Hemoglobin subunit alpha | HBA_MOUSE | 15,085.40 | 100.00% | 3 | 3 | 6 | 38.70% |
| 3 | Hemoglobin subunit alpha | HBA_MOUSE | 15,085.40 | 99.70% | 2 | 2 | 3 | 15.50% |
| 4 | Hemoglobin subunit beta-1 | HBB1_MOUSE | 15,840.20 | 100.00% | 6 | 7 | 22 | 57.80% |
| 5 | Hemoglobin subunit alpha | HBA_MOUSE | 15,085.40 | 100.00% | 5 | 7 | 16 | 56.30% |
| 6 | Hemoglobin subunit alpha | HBA_MOUSE | 15,085.40 | 100.00% | 5 | 6 | 11 | 56.30% |
| 7 | Hemoglobin subunit alpha | HBA_MOUSE | 15,085.40 | 100.00% | 4 | 6 | 11 | 35.20% |
| 8 | Cytochrome b-c1 complex subunit 7 | QCR7_MOUSE | 13,528.30 | 99.80% | 2 | 2 | 4 | 16.20% |
| 9 | Myoglobin | MYG_MOUSE | 17,070.40 | 100.00% | 11 | 16 | 34 | 60.40% |
| 10 | Myoglobin | MYG_MOUSE | 17,070.40 | 100.00% | 4 | 6 | 12 | 22.10% |
| 11 | Nucleoside diphosphate kinase B | NDKB_MOUSE | 17,363.30 | 99.80% | 2 | 4 | 7 | 20.40% |
| 12 | Unidentified | - | - | - | - | - | - | - |
| 13 | Nucleoside diphosphate kinase B | NDKB_MOUSE | 17,363.30 | 99.80% | 2 | 4 | 6 | 20.40% |
| 14 | Alpha-crystallin B chain | CRYAB_MOUSE | 20,069.40 | 100.00% | 2 | 2 | 3 | 10.90% |
| 15 | Alpha-crystallin B chain | CRYAB_MOUSE | 20,069.40 | 100.00% | 2 | 2 | 3 | 21.10% |
| 16 | Transgelin-2 | TAGL2_MOUSE | 22,395.80 | 100.00% | 3 | 3 | 5 | 20.10% |
| 17 | Flavin reductase (NADPH) | BLVRB_MOUSE | 22,196.70 | 99.80% | 2 | 3 | 5 | 8.74% |
| 18 | Glutathione S-transferase Mu 1 | GSTM1_MOUSE | 25,971.90 | 100.00% | 6 | 6 | 12 | 18.80% |
| 19 | 3-hydroxyacyl-CoA dehydrogenase type-2 | HCD2_MOUSE | 27,418.70 | 89.40% | 1 | 2 | 8 | 6.51% |
| 20 | Protein NipSnap homolog 2 | NIPS2_MOUSE | 32,933.60 | 100.00% | 3 | 4 | 6 | 10.70% |
| 21 | Phosphoglycerate mutase 2 | PGAM2_MOUSE | 28,827.80 | 100.00% | 4 | 5 | 8 | 17.40% |
| 21 | Enoyl-CoA delta isomerase 1, mito | ECI1_MOUSE | 32,079.20 | 99.80% | 2 | 3 | 6 | 6.23% |
| 21 | Coiled-coil-helix-coiled-coil protein 3, mitochondrial | CHCH3_MOUSE | 26,334.40 | 100.00% | 3 | 5 | 7 | 11.90% |
| 22 | Succinate dehydrog [ubiquin] iron-sulfur s.u., mito | DHSB_MOUSE | 31,815.00 | 100.00% | 3 | 3 | 6 | 9.57% |
| 22 | Phosphoglycerate mutase 2 | PGAM2_MOUSE | 28,827.80 | 100.00% | 4 | 4 | 7 | 17.40% |
| 23 | Phosphoglycerate mutase 2 | PGAM2_MOUSE | 28,827.80 | 100.00% | 5 | 6 | 10 | 17.80% |
| 24 | Troponin I, cardiac muscle | TNNI3_MOUSE | 24,259.70 | 100.00% | 5 | 7 | 10 | 12.80% |
| 25 | ATP synthase subunit gamma, mitochondrial | ATPG_MOUSE | 32,887.40 | 99.60% | 1 | 1 | 1 | 4.03% |
| 25 | PDZ and LIM domain protein 5 | PDLI5_MOUSE | 63,298.10 | 99.80% | 2 | 3 | 4 | 4.57% |
| 26 | Isocitrate dehydrogenase [NADP], mitochondrial | IDHP_MOUSE | 50,907.20 | 100.00% | 4 | 5 | 8 | 11.30% |
| 27 | Isocitrate dehydrogenase [NADP], mitochondrial | IDHP_MOUSE | 50,907.20 | 100.00% | 6 | 8 | 15 | 14.20% |
| 27 | Elongation factor Tu, mitochondrial | EFTU_MOUSE | 49,508.90 | 100.00% | 8 | 10 | 15 | 17.90% |
| 28 | Beta-enolase | ENOB_MOUSE | 47,025.90 | 100.00% | 9 | 11 | 19 | 25.10% |
| 29 | Isocitrate dehydrogenase [NADP], mitochondrial | IDHP_MOUSE | 50,907.20 | 100.00% | 6 | 8 | 14 | 14.20% |
| 29 | Fumarate hydratase, mitochondrial | FUMH_MOUSE | 54,357.10 | 100.00% | 5 | 7 | 11 | 15.60% |
| 29 | Beta-enolase | ENOB_MOUSE | 47,025.90 | 100.00% | 6 | 9 | 14 | 14.30% |
| 30 | Isocitrate dehydrogenase [NADP], mitochondrial | IDHP_MOUSE | 50,907.20 | 100.00% | 6 | 10 | 18 | 14.20% |
| 31 | NADH dehydrog [ubiquin] flavopr 1, mitochondrial | NDUV1_MOUSE | 50,834.60 | 100.00% | 5 | 6 | 10 | 12.30% |
| 31 | Isocitrate dehydrogenase [NADP], mitochondrial | IDHP_MOUSE | 50,907.20 | 100.00% | 4 | 5 | 7 | 11.50% |
| 32 | Dihydrolipoyl dehydrogenase, mitochondrial | DLDH_MOUSE | 54,272.40 | 100.00% | 4 | 5 | 6 | 8.84% |
| 32 | Pyruvate kinase isozymes M1/M2 | KPYM_MOUSE | 57,845.60 | 100.00% | 4 | 4 | 6 | 8.29% |
| 33 | Catalase | CATA_MOUSE | 59,795.90 | 100.00% | 5 | 5 | 8 | 12.30% |
| 33 | Methylcrotonoyl-CoA carboxyl β chain, mito | MCCB_MOUSE | 61,380.00 | 100.00% | 4 | 4 | 6 | 9.59% |
| 34 | Dihydrolipoyl dehydrogenase, mitochondrial | DLDH_MOUSE | 54,272.40 | 100.00% | 3 | 4 | 6 | 5.89% |
| 34 | Catalase | CATA_MOUSE | 59,795.90 | 100.00% | 4 | 4 | 7 | 10.40% |

Supplementary Table 2. All identified spots pI 6-9

| No. | Protein name | UniProt ID | Mw [2] | Protein identification probability | Number of unique peptides | Number of unique spectra | Number of total spectra | Percentage sequence coverage |
| --- | --- | --- | --- | --- | --- | --- | --- | --- |
| 1 | 10 kDa heat shock protein, mitochondrial | CH10_MOUSE | 10,962.8 | 100.0% | 4 | 4 | 6 | 33.3% |
| 2 | Hemoglobin subunit beta-1 | HBB1_MOUSE | 15,840.2 | 100.0% | 4 | 5 | 11 | 34.7% |
| 3 | Hemoglobin subunit beta-1 | HBB1_MOUSE | 15,840.2 | 100.0% | 9 | 12 | 25 | 74.1% |
| 4 | NADH dehydrog [ubiquinone] 1 alpha subcomplex s.u. 5 | NDUA5_MOUSE | 13,360.2 | 100.0% | 7 | 14 | 24 | 33.6% |
| 5 | Alpha-crystallin B chain | CRYAB_MOUSE | 20,069.4 | 100.0% | 3 | 4 | 6 | 25.7% |
| 6 | Myosin-6 | MYH6_MOUSE | 223,571.2 | 100.0% | 9 | 12 | 21 | 4.0% |
| 7 | Triosephosphate isomerase | TPIS_MOUSE | 32,191.3 | 100.0% | 12 | 16 | 26 | 47.5% |
| 8 | Enoyl-CoA hydratase, mitochondrial | ECHM_MOUSE | 31,475.3 | 100.0% | 9 | 13 | 23 | 31.0% |
| 9 | Hydroxyacyl-coenzyme A dehydrogenase, mitochondrial | HCDH_MOUSE | 34,464.7 | 100.0% | 11 | 14 | 21 | 24.2% |
| 10 | Myozenin-2 | MYOZ2_MOUSE | 29,762.5 | 100.0% | 5 | 7 | 12 | 22.3% |
| 10 | Four and a half LIM domains protein 2 | FHL2_MOUSE | 32,072.2 | 100.0% | 9 | 9 | 16 | 35.8% |
| 11 | Malate dehydrogenase, cytoplasmic | MDHC_MOUSE | 36,512.1 | 100.0% | 7 | 8 | 14 | 26.9% |
| 12 | Pyruvate dehydrogenase E1 component subunit beta, mito | ODPB_MOUSE | 38,937.0 | 100.0% | 4 | 5 | 10 | 13.1% |
| 12 | Malate dehydrogenase, cytoplasmic | MDHC_MOUSE | 36,512.1 | 100.0% | 6 | 7 | 16 | 24.9% |
| 13 | Isocitrate dehydrogenase [NAD] subunit alpha, mito | IDH3A_MOUSE | 39,639.4 | 100.0% | 8 | 11 | 16 | 24.3% |
| 14 | Isocitrate dehydrogenase [NAD] subunit alpha, mito | IDH3A_MOUSE | 39,639.4 | 100.0% | 7 | 8 | 12 | 20.5% |
| 15 | Acetyl-CoA acetyltransferase, mitochondrial | THIL_MOUSE | 44,816.4 | 100.0% | 11 | 20 | 30 | 31.1% |
| 16 | Beta-enolase | ENOB_MOUSE | 47,025.9 | 100.0% | 15 | 18 | 29 | 31.6% |
| 17 | Beta-enolase | ENOB_MOUSE | 47,025.9 | 100.0% | 18 | 19 | 29 | 37.3% |
| 18 | Elongation factor Tu, mitochondrial | EFTU_MOUSE | 49,508.9 | 100.0% | 9 | 11 | 17 | 19.2% |
| 18 | Pyruvate dehydrog E1 component s.u. α, somatic, mito | ODPA_MOUSE | 43,232.5 | 100.0% | 10 | 12 | 17 | 29.2% |
| 18 | Isocitrate dehydrogenase [NADP], mitochondrial | IDHP_MOUSE | 50,907.2 | 100.0% | 6 | 8 | 15 | 13.7% |
| 19 | Beta-enolase | ENOB_MOUSE | 47,025.9 | 100.0% | 18 | 24 | 42 | 42.2% |
| 20 | Beta-enolase | ENOB_MOUSE | 47,025.9 | 100.0% | 18 | 24 | 45 | 39.6% |
| 21 | Beta-enolase | ENOB_MOUSE | 47,025.9 | 100.0% | 7 | 9 | 15 | 16.6% |
| 22 | Beta-enolase | ENOB_MOUSE | 47,025.9 | 100.0% | 15 | 17 | 29 | 44.5% |
| 23 | Beta-enolase | ENOB_MOUSE | 47,025.9 | 100.0% | 15 | 19 | 30 | 37.6% |
| 24 | Alpha-enolase | ENOA_MOUSE | 47,141.7 | 100.0% | 8 | 8 | 15 | 26.5% |
| 25 | Beta-enolase | ENOB_MOUSE | 47,025.9 | 100.0% | 17 | 21 | 36 | 31.6% |
| 26 | Beta-enolase | ENOB_MOUSE | 47,025.9 | 100.0% | 11 | 15 | 23 | 23.0% |
| 27 | Alpha-enolase | ENOA_MOUSE | 47,141.7 | 100.0% | 6 | 7 | 13 | 24.0% |
| 28 | ATP synthase subunit alpha, mitochondrial | ATPA_MOUSE | 59,754.1 | 100.0% | 8 | 8 | 12 | 15.9% |
| 28 | Tripartite motif-containing protein 72 | TRI72_MOUSE | 52,816.4 | 100.0% | 6 | 7 | 11 | 12.8% |
| 29 | ATP synthase subunit alpha, mitochondrial | ATPA_MOUSE | 59,754.1 | 100.0% | 12 | 13 | 20 | 24.6% |
| 30 | ATP synthase subunit alpha, mitochondrial | ATPA_MOUSE | 59,754.1 | 100.0% | 9 | 9 | 15 | 19.0% |
| 30 | UTP--glucose-1-phosphate uridylyltransferase | UGPA_MOUSE | 56,981.2 | 100.0% | 8 | 10 | 16 | 15.7% |
| 31 | UTP--glucose-1-phosphate uridylyltransferase | UGPA_MOUSE | 56,981.2 | 100.0% | 6 | 11 | 17 | 12.0% |
| 31 | ATP synthase subunit alpha, mitochondrial | ATPA_MOUSE | 59,754.1 | 100.0% | 8 | 9 | 14 | 15.6% |
| 32 | Pyruvate kinase isozymes M1/M2 | KPYM_MOUSE | 57,845.6 | 100.0% | 12 | 14 | 21 | 25.6% |
| 32 | Dihydrolipoyl dehydrogenase, mito | DLDH_MOUSE | 54,272.4 | 100.0% | 9 | 11 | 16 | 20.8% |
| 33 | Very long-chain specific acyl-CoA dehydrogenase, mito | ACADV_MOUSE | 70,877.0 | 100.0% | 13 | 18 | 28 | 20.4% |
| 33 | Electron transfer flavoprotein-ubiquin oxidored, mito | ETFD_MOUSE | 68,091.8 | 100.0% | 9 | 11 | 19 | 17.4% |
| 33 | Prelamin-A/C | LMNA_MOUSE | 74,238.9 | 100.0% | 13 | 14 | 22 | 17.0% |

Supplementary Table 3. All identified spots pI 4-7

| Metabolite |  |  |  |  | WT(n=6) | SE | Medium (n=6) | SE | High (n=6) | SE | TTEST |  |  |
| --- | --- | --- | --- | --- | --- | --- | --- | --- | --- | --- | --- | --- | --- |
|  |  |  |  |  |  |  |  |  |  |  | WT/M | M/H | WT/H |
| Sphingolipid | 1 | 5.935 | 5.875 |  | 5.51 | 0.17 | 5.21 | 0.13 | 4.83 | 0.22 | 0.1881 | 0.1840 | **0.0369** |
| Unsaturated | 2 | 5.451 | 5.285 |  | 427.49 | 18.91 | 407.92 | 11.97 | 427.78 | 11.27 | 0.4061 | 0.2550 | 0.9898 |
| glycerol backbone | 3 | 5.252 | 5.181 |  | 41.87 | 1.22 | 41.48 | 0.91 | 44.73 | 0.82 | 0.8029 | **0.0242** | 0.0857 |
| TG | 4 | 4.294 | 4.271 |  | 6.79 | 1.05 | 5.82 | 0.29 | 5.13 | 0.21 | 0.4083 | 0.0865 | 0.1756 |
| Phosphatidylcholine | 5 | 3.369 | 3.311 |  | 170.36 | 4.87 | 169.50 | 3.49 | 187.36 | 3.41 | 0.8879 | **0.0044** | **0.0188** |
| Phosphatidylethanolamine | 6 | 3.174 | 3.106 |  | 24.79 | 1.17 | 25.28 | 0.72 | 26.87 | 0.70 | 0.7287 | 0.1453 | 0.1653 |
| total CH2 | 7 | 1.402 | 1.198 |  | 1778.18 | 67.48 | 1711.80 | 38.27 | 1800.93 | 29.80 | 0.4174 | 0.0978 | 0.7669 |
| C19 cholesterol | 8 | 1.019 | 0.999 |  | 20.53 | 0.24 | 19.40 | 0.26 | 19.82 | 0.34 | **0.0092** | 0.3450 | 0.1252 |
| CH=CHCH2CH3 | 9 | 0.995 | 0.952 |  | 60.37 | 1.96 | 56.25 | 1.40 | 55.61 | 1.72 | 0.1216 | 0.7792 | 0.0986 |
| C21 cholesterol | 10 | 0.93 | 0.905 |  | 22.31 | 0.24 | 21.49 | 0.27 | 22.07 | 0.40 | **0.0469** | 0.2548 | 0.6244 |
| total CH3 | 11 | 0.906 | 0.852 |  | 311.70 | 9.76 | 300.09 | 6.25 | 316.21 | 4.59 | 0.3443 | 0.0670 | 0.6885 |
| C18 cholesterol | 12 | 0.696 | 0.657 |  | 13.97 | 0.23 | 13.47 | 0.31 | 14.21 | 0.19 | 0.2308 | 0.0761 | 0.4485 |
| TMS satellite | 13 | 0.097 | 0.073 |  | 2.39 | 0.12 | 2.15 | 0.06 | 1.88 | 0.09 |  |  |  |
| TMS | 14 | 0.019 | -0.022 | 12 | 385.08 | 22.22 | 346.38 | 7.61 | 295.86 | 15.42 |  |  |  |

Supplementary Table 4. Lipid metabolites quantitated by ^1^H-NMR. Apparent concentration μmol/g tissue. Not corrected for number of protons. Values in bold correspond to statistically significant changes (p < 0.05).

| Transcript name | Sense oligo | Antisense oligo | Nucleotide ID |
| --- | --- | --- | --- |
| 36B4 | 5’AGATTCGGGATATGCTGTTGG3’ | 5’TCGGGTCCTAGACCAGTGTTC-3’ | NM_007475 |
| Rcan1 | 5’GAGACCAGGGCCAAATTT3’ | 5’AGATAAGGGGTTGCTGA3’ | NM_019466.4 |
| FoxO1 | 5’GGGGCAACCTGTCGTACGCCGA3’ | 5’GGCGAATTGAATTCTTCCA3’ | NM_019739.3 |

Supplementary Table 5. Oligonucleotides used in qRT-PCR were designed to span intron-exon boundaries and had amplicon size up to 120bp.

Supplementary Figure 1


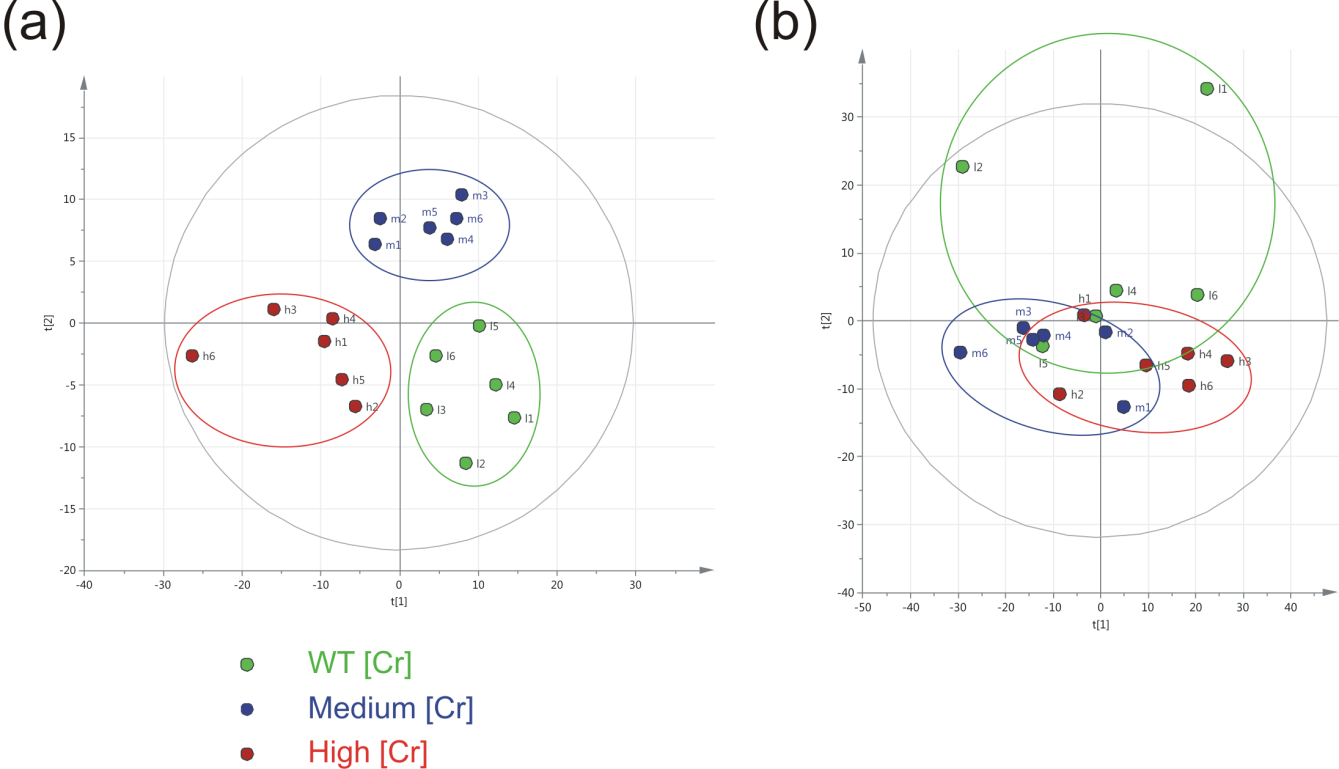


Principal component analysis of ^1^H-NMR spectra for aqueous (A) and lipid (B) metabolites. Coloured dots correspond to WT (‘l’ for low [Cr]; green), medium (‘m’ for medium [Cr]; blue) and high (‘h’ for high [Cr]; red) creatine groups (n=6 each). PLS-DA is a supervised PCA.
